# Correlates and Barriers of Exercise, Stress, and Wellness in Medical Students

**DOI:** 10.1007/s40670-024-02134-5

**Published:** 2024-08-09

**Authors:** McKayla Deisz, Cassie Papproth, Emily Ambler, Margaret Glick, Cassie Eno

**Affiliations:** Creighton University School of Medicine, Omaha, NE USA

**Keywords:** Medical education, Medical student, Exercise, Wellness, Stress

## Abstract

Medical students report higher stress and increased mental illness than peers of similar age. Burnout and depression among medical students and physicians have also been correlated with increased risk of medical errors and decreased importance of altruistic values, such as providing care to medically underserved populations. This study works to analyze the effects of exercise, nutrition, sleep, and other factors affecting wellness and performance of medical students, as well as identifying barriers to exercise and possible solutions. A survey was distributed to medical students in the Midwest to evaluate exercise habits, dietary intake, sleep, and other factors affecting wellness. An investigation of the correlation of variables and comparison of year in school for the variables was conducted. Satisfaction with social relationship and performance in school correlated with exercise, stress, and sleep variables. Several variables, including satisfaction with exercise, social life, and performance in school, showed significant differences based on year in school. No significant findings were noted between students from the various medical institutions at which the survey was distributed regarding satisfaction with exercise, academic performance, social life, personal relationships, hours spent in clerkship/class, hours spent studying, and stress. Students tended to have higher satisfaction with school and social relationships if they engaged in exercise more often, exercised with greater intensity, and slept better. Students identified barriers to exercise including cost, availability, and lack of time. Potential solutions include a workout facility in school buildings/hospitals, allocated wellness periods for exercise, and a more consistent schedule.

**Supplementary Information:**

The online version contains supplementary material available at 10.1007/s40670-024-02134-5.

## Introduction

Medical school can be challenging for students due to the demanding course rigor and high standard of performance. Many students across the country struggle with maintaining wellness and living a balanced life during their education, often prioritizing their coursework over healthy lifestyle choices, including exercise and sleep [1, 2]. Depression rates for medical students are higher compared to those for their peers in graduate school or young adults in the workforce; specifically, 21.2% of medical students reported depression compared to 8–15% for other young adults [3]. The rates of depression for residents are between 27 and 35%, and at least 6% of all medical trainees considered suicide at some point during their training [4]. Burnout fatigue is reported in 45–71% of medical students, and in greater than 50% of residents and physicians [5–8]. These burnout and depression among medical students and physicians have been shown to be correlated with increased risk of medical errors, decreased importance of altruistic values (i.e., being less likely to provide care to medically underserved populations), and reductions in overall professional work effort [7, 8, 10]. Another study found that matriculating medical students had lower rates of depression and a higher reported quality of life compared to similar age college graduates [11]. This demonstrates that while students enter medical school with better mental health than their non-medical counterparts, at some point during training they suffer a drastic decline in mental health to below that of their peers. Factors such as excessive workload, struggles with time management/work life balance, and health concerns are some examples of the many stressors students face [12]. This increased stress can predispose them to poorer mental health outcomes and burnout as they progress in their careers. Although stress and burnout are separate entities, greater levels of stress are shown to decrease resiliency to burnout [13]. Medical institutions need to be cognizant of this trend and engage in active strategies to assess stress and burnout as well as ensure students can manage their stress through healthy coping mechanisms.

One such mechanism is exercise, which has been shown to improve physical and mental health and reduce symptoms of anxiety and depression [14]. Many studies have shown the positive effects of exercise on mental health, indicating that exercise leads to a variety of physiological changes that improve self-esteem and mood while decreasing stress and anxiety levels [15]. Other studies have demonstrated the efficacy of exercise in treating symptoms of mild depression and the association between regular exercise and lower depression and anxiety rates [16]. In medical students specifically, physical activity has been found to be negatively correlated with burnout and positively correlated with improved quality of life [17–19]. In residents and fellows at one institution, increased physical activity through an incentivized exercise program was correlated with increased reported quality of life [20]. Other studies have shown that increased physical activity is associated with higher professional efficacy, and decreased exercise frequency was correlated with lower professional efficacy [2, 21]. These studies demonstrate the importance of exercise for those in a stressful environment which predisposes individuals to higher risk of mental health issues. Additionally, there does not seem to be a correlation between a certain type of exercise and the degree of effect it has on mood; either aerobic or anaerobic exercise can bring about a comparable improvement in mental health and wellness [15]. Furthermore, a study on medical students at one US institution found that increased exercise was correlated with increased probability of the student perceiving lower stress levels [22]. Overall, engaging in physical activity, especially with increased intensity, has a positive effect and should be strongly encouraged in medical school.

Sleep is another factor that can have a significant impact on stress. At one American medical school, inadequate sleep was correlated with lower professional efficacy and higher exhaustion scores, and pathological sleepiness was correlated with higher reported burnout levels [2]. In a study of college students, poor sleep quality along with increased sleep onset latency, nighttime wakings, and daytime sleepiness were positively associated with depressive symptoms [23]. In another study among medical students in Saudi Arabia, sleep quality and stress were found to be negatively correlated. Students that scored higher on a stress index scale were significantly more likely to have poor quality sleep, and vice versa [24]. Sleep and stress seem to have an inverse relationship that also impacts wellness and burnout, so students should be strongly encouraged to get sufficient sleep each night to reduce their perceived stress.

By creating habits that promote health, such as regular exercise and sufficient sleep, students can set themselves up for success, create long-lasting healthy habits to prepare for the tough years of residency, and provide a positive example for their patients. A study on general wellness was conducted on Creighton University medical students last year, and the results showed a correlation between satisfaction with exercise and mental well-being (Kepple, J. Noel, M. Johnson, R. Kaczynski D. Jackson, B. Madhavan, K PhD. Meyer, C. EdS., unpublished data, February 2022). This research builds on those results to further explore the specific exercise habits, social interactions, and sleep patterns of medical students in various institutions in the midwestern United States.

Many studies have previously analyzed the well-being and stress levels of students. These studies evaluated many different factors, including depression rates, levels of burnout, physical activity, and suicidal ideology [2–5, 8, 12, 17–19, 21, 22, 25]. This study uniquely focused on medical students in the midwestern US to uncover more details about exercise, social factors, and sleeping habits in this specific population. It also explored whether these factors have any relationship with the wellness of students dealing with the stress of medical school. Specifically, relationships between stress levels and exercise, alcohol intake, sleep quantity/quality, personal/social life, and academic satisfaction were analyzed. Barriers that prevent students from engaging in these healthy activities were also evaluated. The results from this study could contribute to future medical school guidelines, both formal and informal, to enhance wellness in medical students and help them develop positive coping mechanisms to address stress throughout their education, residency, and career.

## Methods

### Data Collection and Study Population

This study collected data on exercise habits, sleep, and other various factors affecting wellness among medical students. A survey was designed to assess these factors for each student in a uniform and brief manner to encourage participation. The survey was distributed to all medical students (M1–M4 years) at medical institutions across the Midwest that agreed to participate in this study. Medical institutions that approved survey distribution to their students include Creighton University School of Medicine (both the Omaha and Phoenix campuses), The University of South Dakota, The University of Nebraska Medical Center, Rush Medical College, and Southern Illinois University. This study was approved by the Institutional Review Board of Creighton University.

### Description of Survey

The survey assessed students’ perception of their current habits including exercise, eating habits, emotional well-being, academic performance, and stress. Eating habits included diet, alcohol consumption, and caffeine consumption. Exercise habits included type of exercise, number of hours exercised per week, comparison of exercise to before beginning medical school, intensity of exercise, and satisfaction with exercise. Stress included sleep habits and stress level. Emotional well-being evaluated social life and personal relationships. Academic performance was evaluated with satisfaction of performance, number of hours spent studying, and number of hours spent attending class or clerkships. The survey also inquired about any barriers students may face regarding exercise, healthy eating, and sufficient sleep. Additional questions addressed age, gender, race, ethnicity, year in school, medical college currently attending, and barriers to exercise. The survey utilized both multiple choice and qualitative questions on a 5-point Likert scale commensurate to the question (see Appendix for full survey).

### Statistical Analysis

Primary variables collected from the survey include type of exercise, number of hours per week spent exercising, exercise intensity, satisfaction with exercise, satisfaction with academic performance, satisfaction with social life, satisfaction with personal relationships, diet, sleep quality, number of caffeinated beverages consumed in a week, number of alcoholic beverages consumed per week, type of diet, number of hours spent in clerkship or class, and number of hours spent studying. Descriptive statistics were employed to describe the characteristics of the sample. Barriers and solutions to exercise were open-ended questions. These questions were coded for themes found in the reported answers.

Series of ANOVAs were run to test for differences between institutions and class year (i.e., M1, M2, M3, M4). Correlation analyses were run to examine the relationship between exercise and the following factors:

1. Wellness (e.g., satisfaction with exercise, social life, personal relationships)
2. Stress (e.g., level of stress, sleep quality)
3. Nutrition (e.g., type of diet, number of alcoholic and caffeinated beverages consumed)
4. Barriers to exercise (e.g., lack of time, transportation, space, financials)

These factors were also compared between year in school and across medical institution.

## Results

### Demographics

A total of 393 students from five participating medical schools completed the survey. The overall response rate was not possible to calculate because all participating medical schools requested that the email was sent by a school representative rather than the investigators. For this reason, the total number of students that the survey was distributed to is not available. Demographic information on the sample of the 393 students that completed the survey is shown in Table 1; demographic information was not completed by all the students.

**Table 1:** Characteristics of the sample (*N* = 393)*

Characteristic	*N* (%)	Range
Age, mean (SD)	25.44 (2.64)	21–43
Gender		
Male	35.1	
Female	60.1	
Other	1.0	
Race		
White	78.9	
Black	1.0	
American Indian	0.5	
Asian	8.9	
Other	2.3	
Prefer not to say	1.5	
Ethnicity		
Spanish/Hispanic/Latino origin	5.6	
Not Spanish/Hispanic/Latino origin	90.6	
Year in school		
M1	10.9	
M2	33.8	
M3	28.5	
M4	23.2	
Institution		
Creighton University School of Medicine	54	
Southern Illinois University School of Medicine	6.6	
Rush Medical College	1.3	
University of South Dakota Sanford School of Medicine	12.7	
University of Nebraska Medical Center	15	

*Totals may not add to 100% because of missing data

### Averages

On average, students reported 5.7 h of exercise per week. They rated the intensity of the exercise as moderate and endorsed having little satisfaction with their exercise habits. In general, they reported currently engaging in less exercise than prior to medical school. Additionally, students reported an average of 3.39 alcohol drinks and 2.06 cups of caffeinated beverage consumed in a week. They rated their average stress level in a normal week as moderate and average quality of sleep as good. Regarding schoolwork, students spent an average of 35.45 h attending class or clerkships, and an average of 24.08 h studying. They rated their satisfaction with performance in school, social life, and personal relationships as moderately satisfied (see Tables 2 and 3 for means and SDs).

**Table 2:** Average responses on survey constructs (average number of hours/drinks)

	EXHR	CLASS	STUDY	ALC	ENG
Average	5.7	35.45	24.08	3.39	2.06
SD	5.36	16.92	15.28	3.36	1.8

EXHR: During an average school week, how many hours do you exercise?—Hours per week

CLASS: How many hours do you spend attending class/clerkships per week?—Hours in class

STUDY: How many hours do you spend studying in an average school week?—Hours studying

ALC: How many alcoholic drinks do you consume in a week?—Number of drinks:

ENG: How many cups of energy drinks (coffee included) do you have in a day?—Number of drinks

**Table 3:** Average responses on survey constructs (5-point Likert scale)

	EXIN	EXSA	EXHA	STR	SLP*	SA PER	SA SOC	SA REL
Average	3.89	2.48	2.34	2.99	2.69	3.35	3.05	3.38
SD	1.17	1.09	1.09	0.76	0.7	0.9	1	1.02

EXIN: Please rate the intensity of your exercise

EXSA: Please rate your satisfaction with your exercise habits in medical school

EXHA: How would you compare your exercise habits to before you began medical school?

STR: Please rate your average level of stress in a normal school week

*SLP: Please rate your quality of sleep during an average school week; 4-point Likert scale

SA PER: Please rate your satisfaction with your performance in school

SA SOC: Please rate your satisfaction with your social life

SA REL: Please rate your satisfaction with your personal relationships

### Between School Comparisons

No significant findings were noted between students from the various medical institutions at which the survey was distributed regarding satisfaction with exercise, academic performance, social life, personal relationships, hours spent in clerkship or class, hours spent studying, and stress. Due to this finding, results will be reported for all schools together.

### Correlation Analysis

The correlations between the measures can be found in Table 4. To begin understanding the complex relationship between exercise and wellness, correlations were run for combinations of the following variables: hours exercised per week, exercise intensity, exercise habits compared to before medical school, alcohol/caffeine consumption, stress, sleep quality, hours spent attending class/clerkship/studying, and satisfaction with exercise/academic performance/social life/personal relationships. Relationships of note include positive correlations between satisfaction with exercise and number of hours exercised per week (*r* = 0.331), exercise intensity (*r* = 0.275), increased exercise compared to before medical school (*r* = 0.121), and satisfaction with academic performance (*r* = 0.213)/social life (*r* = 0.212)/personal relationships (*r* = 0.163) (Table 4, *p* < 0.001). Satisfaction with exercise was negatively correlated with average stress (*r* =  − 0.132) (Table 4, *p* < 0.05).

**Table 4:** Correlation matrix for measures

	EXHR	EXIN	EXSA	EXHA	DIET	ALC	ENG	STR	SLP	CLASS	STUDY	SAPER	SASOC	SAREL
EXHR	1													
EXIN	0.275**	1												
EXSA	0.331**	0.522**	1											
EXHA	0.121*	0.296**	0.607**	1										
DIET	0.208**	0.097	0.056	− 0.028	1									
ALC	0.007	0.147**	0.057	0.044	− 0.095	1								
ENG	0.088	0.023	0.036	− 0.046	0.092	0.231**	1							
STR	0.031	− 0.099	− 0.132*	− 0.094	0.110*	− 0.095	0.216**	1						
SLP	− 0.080	0.071	0.048	0.029	− 0.062	− 0.020	− 0.123*	− 0.342**	1					
CLASS	0.082	− 0.052	− 0.077	− 0.086	0.086	− 0.042	0.150**	0.042	− 0.053	1				
STUDY	0.075	− 0.038	− 0.027	− 0.087	− 0.046	− 0.097	0.010	0.198**	− 0.105*	− 0.403**	1			
SAPER	− 0.021	0.106*	0.213**	0.207**	− 0.060	0.000	− 0.046	− 0.299**	0.180**	− 0.084	− 0.065	1		
SASOC	− 0.035	0.142**	0.212**	0.256**	− 0.067	0.201**	− 0.073	− 0.361**	0.283**	− 0.078	− 0.138**	0.374**	1	
SAREL	− 0.108	0.043	0.163**	0.228**	− 0.011	0.026	− 0.052	− 0.261**	0.184**	− 0.076	− 0.093	0.315**	0.594**	1

*Correlation was statistically significant at *p* < 0.05

**Correlation was statistically significant at *p* < 0.01

EXHR: During an average school week, how many hours do you exercise?—Hours per week

EXIN: Please rate the intensity of your exercise

EXSA: Please rate your satisfaction with your exercise habits in medical school

EXHA: How would you compare your exercise habits to before you began medical school?

DIET: Please select which diet most closely resembles your own.—Selected Choice

ALC: How many alcoholic drinks do you consume in a week?—Number of drinks:

ENG: How many cups of energy drinks (coffee included) do you have in a day?—Number of drinks

STR: Please rate your average level of stress in a normal school week

SLP: Please rate your quality of sleep during an average school week

CLASS: How many hours do you spend attending class/clerkships per week?—Hours in class

STUDY: How many hours do you spend studying in an average school week?—Hours studying

SA PER: lease rate your satisfaction with your performance in school

SA SOC: Please rate your satisfaction with your social life

SA REL: Please rate your satisfaction with your personal relationships

### Between Class Comparisons

Table 5.

**Table 5:** Average response by class and significant differences between classes

	M1 students (class of 2026)	M2 students (class of 2025)	M3 students (class of 2024)	M4 students (class of 2023)	Significant differences
Satisfaction with exercise	2.24	2.5	2.34	2.71	M4 > M1, M3
Satisfaction with academic performance	3.2	3.35	3.06	3.78	M4 > M1, M2, M3 M2 > M3
Satisfaction with social life	2.76	3.12	2.8	3.39	M4 > M1, M2, M3 M2 > M1, M3
Satisfaction with personal relationships	3	3.42	3.23	3.67	M4 > M3, M1 M2 > M1
Hours attending class/clerkship	25.76	21.09	46.57	46.69	M3, M4 > M1, M2 M1 > M2
Hours Studying	34.39	32.02	18.92	13.85	M1, M2 > M3, M4 M3 > M4
Stress	3.37	2.99	2.98	2.81	M1 > M2, M3, M4

*M1* first year medical student, *M2* second year medical student, *M3* third year medical student, *M4* fourth year medical student)

### Satisfaction with Exercise

The overall reported level of exercise satisfaction was 2.48 (between a little satisfied and moderately satisfied) (Table 5). There was a significant increase in exercise satisfaction in M4s compared to M3s and M1s (*F*_(3, 367)_ = 2.750, *p* < 0.05), suggesting that students were more satisfied with their exercise habits in their final year of medical school.

### Satisfaction with Academic Performance

The overall reported level of academic performance satisfaction was 3.35 (between moderately satisfied and very satisfied). There was a significant increase in satisfaction in M4s compared to the other classes, along with a significant increase in M2s compared to M3s (*F*_(3, 357)_ = 11.807, *p* < 0.001). This suggests that fourth year students were more satisfied with their academic performance than other classes, and second years were more satisfied with academic performance than third year students.

### Satisfaction with Social Life

The overall reported level of social life satisfaction was 3.05 (moderately satisfied). There was a significant increase in satisfaction in M4s compared to all other classes, along with a significant increase in M2s compared to M1s and M3s (*F*_(3, 357)_ = 7.801, *p* < 0.001). This suggests that fourth year students were more satisfied with their social life than other classes. Additionally, second year students were more satisfied with their social lives than third and first years.

### Satisfaction with Personal Relationships

The overall reported level of satisfaction in personal relationships was 3.38 (between moderately satisfied and very satisfied). There was a significant increase in satisfaction in M4s compared to M3s and M1s, along with a significant increase in M2s compared to M1s (*F*_(3, 357)_ = 5.374, *p* < 0.01). This indicates that fourth year students are more satisfied with their personal relationships than the third and first years, and the second year students are more satisfied with their personal relationships than the first years.

### Hours Spent Attending Class/Clerkship

The overall average of reported hours spent in class or clerkships was 35.45. M3s and M4s spend a significant number of hours in clerkship/class when compared to M1s and M2s. The M1s also spend more time in class/clerkships than the M2s (*F*_(3, 356)_ = 130.034, *p* < 0.001). This shows that the upperclassmen spend more time in class and clerkships than the underclassmen, and M1s also spend more time in class than the M2s.

### Hours Spent Studying

The overall average of reported hours spent studying was 24.08. M1s, and M2s reported more study hours than the M3s and M4s. Additionally, the M3s reported more study hours than the M4s. There was no significant difference between the M1s and M2s (*F*_(3, 353)_ = 47.734, *p* < 0.001). These findings indicate the underclassman spend more time studying than the upperclassmen, and M3s spend more time studying than M4s.

### Stress Levels

The overall reported level of stress was 2.99, or moderately stressed. There was a significant increase in stress in M1s when compared to all other classes. The findings between the other classes were statistically insignificant (*F*_(3, 356)_ = 5.186, *p* < 0.01). This suggests that first year medical students are more stressed on average than other medical students.

### Non-significant Findings Between Years

There was no significant difference between year in medical school compared to number of hours of exercise per week or intensity of exercise. There were no statically significant differences regarding alcohol of caffeine consumption between years in school. There were also no differences in quality of sleep.

### Barriers to Exercise

**Fig. 1:**
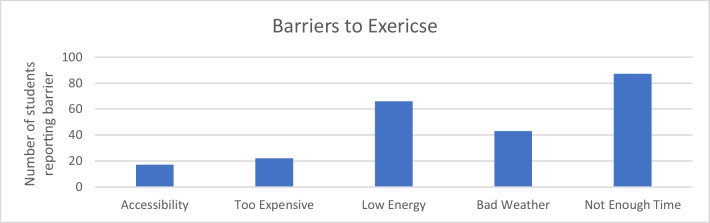
Barriers to exercise

Students identified barriers to exercise as issues with accessibility, cost, energy levels, weather, and time. Lack of time was the most common barrier, with low energy as a close second (see Fig. 1).

### Solutions for Increasing Exercise in Medical Students

**Fig. 2:**
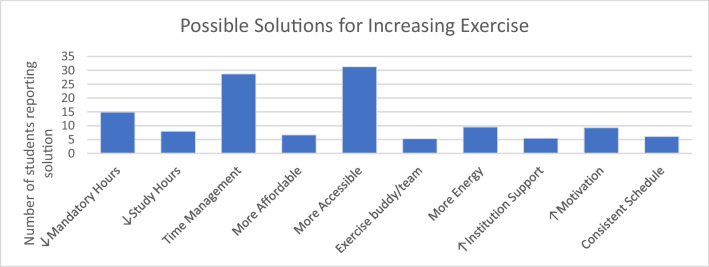
Possible solutions for increasing exercise

Students identified possible solutions for increasing exercise in medical students. These solutions were organized by common topic (Fig. 2). Popular solutions included increasing accessibility to exercise and better time management. Students also answered that fewer mandatory hours would help facilitate more exercise.

## Discussion

### Overall Averages

The students in this study reported an average of 5.7 h/week spent exercising, with an average intensity as moderate. This amount echoes the WHO recommendation of 150–300 min/week at moderate intensity, or 75–150 min/week at high intensity [14]. It should be noted that the standard deviation of hours was 5.36; therefore, there is considerable variability in exercise duration per week. Additionally, students that are exercising more often reported greater intensity of exercise, which would result in these guidelines not being met for those exercising less than 150 h a week at a lower intensity. Increased exercise and duration were both positively associated with satisfaction with exercise, though students overall reported an average of little satisfaction with exercise in medical school.

Students also reported moderate levels of stress along with moderate levels of satisfaction in other aspects of their lives including academic performance, social life, and personal relationships. In this study, no data from non-medical students was collected to compare stress levels directly. However, a meta-analysis of the prevalence of depression among medical students showed that the 12-month prevalence of depression in medical students was between 2.2 and 5.2 times greater than the non-medical students of a similar age range [26]. As stress can be a major contributing factor to depression, perhaps lower levels of stress and increased satisfaction with academics, personal life, and social life could impact the prevalence of depression in medical students. Future research is needed to elucidate the specific relationship between the studied variables and depression.

### Comparisons Between Years in School

#### Satisfaction with Exercise, Academic Performance, Social Life, and Personal Relationships

There was a significant difference regarding exercise satisfaction between the fourth year medical students when compared to the first and third year students. At first, one might attribute this to the increased time first and third year students spend in clerkship/class and studying. Specifically, the survey results showed that the first year students spend significantly more time attending class than the second years and more time studying than third and fourth years. Similarly, the data collected showed that third year students spend more time in class/clerkship than first or second years and more time studying than fourth years. However, despite differences in satisfaction with exercise and hours dedicated to medical school activities, there was no statistically significant difference between exercise intensity or average number of hours exercised per week. Additionally, fourth year students reported higher satisfaction with academic performance and social life than the other three classes and higher satisfaction with personal relationships when compared to the pre-clerkship years. This could be due to a wide variety of factors, including student perception and difference in academic schedules. The fourth year students often have fewer mandatory medical school commitments and exams compared to other students (though this varies by institution), which could give them a sense of more autonomy over their schedule and, subsequently, an increase in satisfaction with the amount of exercise they are able to accomplish (even if the actual number of hours of exercise have not changed). One study has shown that employee-oriented flexible work hours may have benefits on employee wellness [27]. Similarly, perhaps more control over their own schedule (picking electives, reserved time for residency applications, etc.) could account for these differences. Additionally, medical school curricula vary from school to school, so interpretation of this data may differ by institution. More information would need to be collected to further elucidate this pattern.

#### Stress

Medical students have higher rates of anxiety and depression compared to others of similar age, and stress is a major factor than can precipitate these mental health issues [26, 28]. Our findings showed that the M1 students reported significantly more stress than the other classes. However, another study by Niemi et al. demonstrated that stress increased as students progressed through medical school, citing the introduction into the clinical setting in later years of medical education as a possible cause of their findings [29]. Our study had a fewer number of first year students (*N* = 41), which could account for the differences found. Another key difference is that Niemi et al. study was conducted in a 6-year medical program. Perhaps the US model better prepares the students to enter their clerkship years, thereby reducing the stress students feel as they progress through their medical training. If that is true, however, midwestern schools could improve upon their resources and support to first year students to help decrease their stress as they begin medical school. Niemi et al. also found that increased stress at the beginning of training correlated with increased stress later in their training [29]. Medical schools should do all they can to support their new students and reduce stress as it could result in decreased stress in years to come.

### Correlation Analysis

#### Exercise

Exercise satisfaction correlated positively with reported hours exercised per week, intensity of exercise, increased exercise habits compared to before medical school, and satisfaction with academic performance, social life, and personal relationships. These findings could be due to the positive effects of exercise, which have been correlated with increased reported quality of life and shown to provide distraction from negative thoughts, increase self-esteem, and encourage social engagement [15, 17–20, 30]. However, if exercise increases self-esteem, perhaps increased satisfaction with performance in medical school, social life, and personal relationships could be due to increased self-confidence and therefore a difference in perception of satisfaction, not observable improvements in these three areas. Additionally, the results showed that while satisfaction with exercise was positively correlated with these findings, number of hours exercised compared to satisfaction with academic performance, social life, and personal relationships was not significant, and intensity of exercise, while significant compared to satisfaction with academic performance and social life, showed smaller Pearson correlation values. This could imply that the amount/intensity of exercise does not affect satisfaction in these areas as much as personal satisfaction with the exercise one engages in. Alternatively, as these relationships are correlational, it is possible that increased satisfaction with academic performance, social life, and personal relationships results in increased satisfaction with exercise satisfaction. Regardless of amount/type/intensity of exercise, if students perceive their satisfaction to be higher in these areas with increased personal satisfaction with exercise, increasing exercise opportunity and accessibility for medical students could be beneficial for the wellness of medical students.

#### Stress

Increasing stress level was negatively correlated with hours slept, satisfaction with exercise, satisfaction with academic performance, satisfaction with social life, and satisfaction with personal relationships. Several other studies have also demonstrated relationships between these factors. A meta-analysis on various randomized control trials suggested that exercise significantly decreased anxiety symptoms [31]. Another study showed that patients with depression had reduced volume of brain structures, including the hippocampus, yet in response to exercise and increased physical health, these regions demonstrated plasticity [32]. Rao et al. found that adding aerobic exercise to the treatment of depression, anxiety, and self-esteem produced improvements in all three factors and overall quality of life [33]. Additionally, one study on medical student wellness found that stress had a significant negative relationship with performance on medical school exams [25]. These studies demonstrate the effects of exercise on stress, quality of life, and brain structure, implying that there is likely a causal effect from increased satisfaction with exercise resulting in decreased stress, which in turn may lead to increased satisfaction in other aspects of life, including academic performance.

#### Sleep

Positive correlations were found between higher quality of sleep and satisfaction with academic performance, social life, and personal relationships. There was no correlation between increased quality of sleep and satisfaction with exercise, number of hours exercised, or intensity of exercise. This implies that there are other relationships between sleep, stress, and satisfaction in academics/social life/personal relationships that are unrelated to exercise. Increased periods of sleep deprivation have been shown to induce increased anxiety [34]. Pathological sleepiness and sleeping less than 7 h a night were independent predictors of burnout at one medical institution in the USA [2]. Another study stated that poor sleep is likely a contributing factor for mental health difficulties, including depression, anxiety, and stress [35]. However, a separate study showed that some aspects of sleep and mental health were bi-directional, and that certain factors, such as increased friendship quality and higher academic achievement, had unidirectional positive effects on sleep quality [36]. Sleep, stress, and overall well-being clearly have a complex causal relationship, but it is apparent that they are closely related. Knowing that higher quality of sleep and increased satisfaction in various life aspects are related, adequate sleep time, and proper sleep hygiene must be considered of utmost importance for well-being in medical students.

#### Barriers and Solutions to Increasing Exercise

Students identified many barriers to exercise, most notably lack of time, low energy, and bad weather. These results are similar to those found in a systematic review of barriers to physical activity in high school and college students where the main barriers identified in this study were lack of time, motivation, and accessibility [37]. These results show that barriers to exercise are similar across academic institutions and are not unique to medical schools. It would make sense that medical students, who likely have a more rigorous course load and higher expectations than high school or college students, would suffer from the same barriers, but perhaps with higher consequences, as demonstrated by the higher rates of mental illness and burnout [4–7]. Another study found that the positive outcomes of exercise were also the barriers to exercise. For example, low mood and stress prevented people from exercising when their reason for exercising was to elevate mood and decrease stress [38]. This phenomenon could apply to medical students as well. Although low mood was not a commonly reported barrier to exercise, students identified low energy as a problem, and mood and energy can affect one another. Additionally, the Midwest can have colder temperatures than other areas of the USA where students might have the opportunity to exercise outside year-round. In the systematic review conducted by Silva et al., multiple studies identified weather (especially cold weather) as a barrier to exercise [37].

Accessibility was another commonly reported barrier. While it was not as popular of a choice as lack of time, low energy, or bad weather, it was interestingly the most identified solution of the barriers to exercise, followed closely by improved time management. These two factors influence each other; lack of access to a nearby, safe, and affordable workout space can result in students having to travel further for such a space, therefore making time management and balancing studying and mandatory commitments more difficult. Some potential options to address these issues could include forming partnerships with nearby health organizations to offer student discounts, providing dedicated time for student exercise, and holding health events such as strength training, yoga classes, or even health and wellness seminars that would count towards continuing education or other school requirements. Additionally, school advisors could be more available to help students create schedules and balance their workload more efficiently, as they self-identified time management as the main barrier they face to exercise. This would be a great opportunity for a future study to examine benefits of supporting students to develop better time management skills. Finally, a medical school in Europe implemented an exercise program into the curriculum to give students a time and place to exercise weekly [39]. Other institutions could implement similar programs to support students and provide more opportunity for exercise in the otherwise hectic schedule of medical students.

The next most identified solution was decreased mandatory hours. However, hours spent attending class demonstrated no significant relationships with time spent exercising, hours of sleep, or satisfaction with exercise, academic performance, social life, or personal relationships. One study showed that increased work hours does not lead to depression [40]. Students might feel that they need more free time to increase exercise hours, but perhaps it is more important to focus on improving time management skills and increasing access to affordable and nearby workout facilities. Alternatively, another study showed that increased work engagement in medical students was negatively correlated with burnout and perceived stress [41]. One study exploring medical student perceptions found that students engaged most with student-initiated activities [42]. Perhaps in combination with improved time management skills and access to exercise, students and institutions should prioritize engagement and student lead activities to better support student wellness.

## Limitations

This study included a sample of medical students during all 4 years of medical school from six different medical institutions. However, there are some notable limitations, including number of students from different schools, different years in school, difference in time of year that the survey was distributed, and limit of the study to the Midwest, which may result in different barriers (e.g., weather) than other locations.

## Final Conclusions

Wellness of the mind, body, and spirit in future physicians is of utmost importance. Currently, medical students suffer from more mental health issues, including anxiety and depression, than their peers. This is unacceptable. Exercise, diet, and sleep are key factors in student well-being. This study showed that increased exercise satisfaction was correlated with decreasing stress and improving satisfaction in academic performance, social life, and personal relationships. Additionally, barriers such as lack of time, low energy, and bad weather were identified, with increasing accessibility and time management skills as the most popular potential solutions. More studies should be conducted in this population to further explore the relationships and examine potential causal factors. Further research will investigate barriers to exercise to create possible interventions. These results will have the potential to inform new policies in the Creighton Medical school curriculum and possibly other medical college curriculums throughout the Midwest to encourage student well-being and success.

## Supplementary Information

Below is the link to the electronic supplementary material.

Supplementary file1 (DOCX 23 KB)

## Data Availability

The datasets generated for the current study are not publicly available but are available from the corresponding author on reasonable request.
