# Supplementary material for: Correlates and Barriers of Exercise, Stress, and Wellness in Medical Students
Source: Med Sci Educ. 2024 Aug 9;34(6):1433–44. doi: 10.1007/s40670-024-02134-5 (PMC11699034; doi:10.1007/s40670-024-02134-5)
Supplement: Supplementary file 1 — Supplementary file1 (DOCX 23 KB) [file 40670_2024_2134_MOESM1_ESM.docx]

Wellness Survey:

Q1. Which medical school do you attend?

________________________________________________________________

Q2. What year will you graduate medical school?

- Class of 2023
- Class of 2024
- Class of 2025
- Class of 2026

Q3. How old are you?

|  | 18 | 21 | 24 | 28 | 31 | 34 | 37 | 40 | 44 | 47 | 50 |
| --- | --- | --- | --- | --- | --- | --- | --- | --- | --- | --- | --- |

| Age | 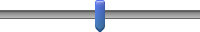 |
| --- | --- |

Q4. Choose one or more races that you consider yourself to be.

- White or Caucasian
- Black or African American
- American Indian/Native American or Alaska Native
- Asian
- Native Hawaiian or Other Pacific Islander
- Other
- Prefer not to say

Q5. Are you of Spanish, Hispanic, or Latino origin?

- Yes
- No

Q6. Please identify which gender most accurately represents you.

- Male
- Female
- Non-binary / third gender
- Prefer not to say

Q7. During an average school week, how many hours do you exercise?

|  | 0 | 10 | 20 | 30 | 40 | 50 | 60 | 70 | 80 | 90 | 100 |
| --- | --- | --- | --- | --- | --- | --- | --- | --- | --- | --- | --- |

| Hours per week: | 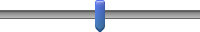 |
| --- | --- |

Q8. What type of exercise do you engage in? Please select all that apply.

- Aerobic exercise (indoors)
- Aerobic exercise (outdoors)
- Weight lifting
- Yoga
- High intensity interval training
- Sporting events
- Other, please specify __________________________________________________

Q9. Please rate the intensity of your exercise.

- No exercise
- Light intensity
- Light to moderate intensity
- Moderate intensity
- Moderate to high intensity
- High intensity

Q10. Please rate your satisfaction with your exercise habits in medical school.

- Not at all satisfied
- A little satisfied
- Moderately satisfied
- Very satisfied
- Extremely satisfied

Q11. How would you compare your exercise habits to before you began medical school?

- I exercise much less often in medical school
- I exercise less often in medical school
- I exercise has not changed since I started medical school
- I exercise more often in medical school
- I exercise way more often in medical school

Q12. Please select which diet most closely resembles your own.

- Standard American Diet
- Vegetarian
- Vegan
- Keto
- Mediterranean
- Other, please specify: __________________________________________________

Q13. How many alcoholic drinks do you consume in a week?

|  | 0 | 10 | 20 | 30 | 40 | 50 | 60 | 70 | 80 | 90 | 100 |
| --- | --- | --- | --- | --- | --- | --- | --- | --- | --- | --- | --- |

| Number of drinks: | 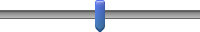 |
| --- | --- |

Q14. How many cups of energy drinks (coffee included) do you have in a day?

|  | 0 | 2 | 4 | 6 | 8 | 10 | 12 | 14 | 16 | 18 | 20 |
| --- | --- | --- | --- | --- | --- | --- | --- | --- | --- | --- | --- |

| Number of drinks | 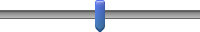 |
| --- | --- |

Q15. Please rate your quality of sleep during an average school week.

- Very restless
- Moderately restless
- Good
- Great

Q16. Please rate your average level of stress in a normal school week.

- 0 - not at all stressed
- 1 - mildly stressed
- 2 - moderately stressed
- 3 - very stressed
- 4 - incredibly stressed

Q17. How many hours do you spend attending class/clerkships per week?

|  | 0 | 10 | 20 | 30 | 40 | 50 | 60 | 70 | 80 | 90 | 100 |
| --- | --- | --- | --- | --- | --- | --- | --- | --- | --- | --- | --- |

| Hours in class | 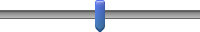 |
| --- | --- |

Q18. How many hours do you spend studying in an average school week?

|  | 0 | 10 | 20 | 30 | 40 | 50 | 60 | 70 | 80 | 90 | 100 |
| --- | --- | --- | --- | --- | --- | --- | --- | --- | --- | --- | --- |

| Hours studying | 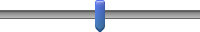 |
| --- | --- |

Q19. Please rate your satisfaction with your performance in school.

- Not at all satisfied
- A little satisfied
- Moderately Satisfied
- Very Satisfied
- Extremely Satisfied

Q20. Please rate your satisfaction with your social life.

- Not at all satisfied
- A little satisfied
- Moderately Satisfied
- Very Satisfied
- Extremely Satisfied

Q21. Please rate your satisfaction with your personal relationships.

- Not at all satisfied
- A little satisfied
- Moderately Satisfied
- Very Satisfied
- Extremely Satisfied

Q22. Please Identify any barriers to exercise that you have experienced.

- Lack of nearby workout space
- Too expensive
- Low energy
- Weather is not conducive to exercising outside
- Not enough time with other responsibilities
- Feeling ill
- Other, please specify __________________________________________________

Q23. Please identify any solutions that would allow you to increase your satisfaction with the amount of time you spend exercising per week.

________________________________________________________________
